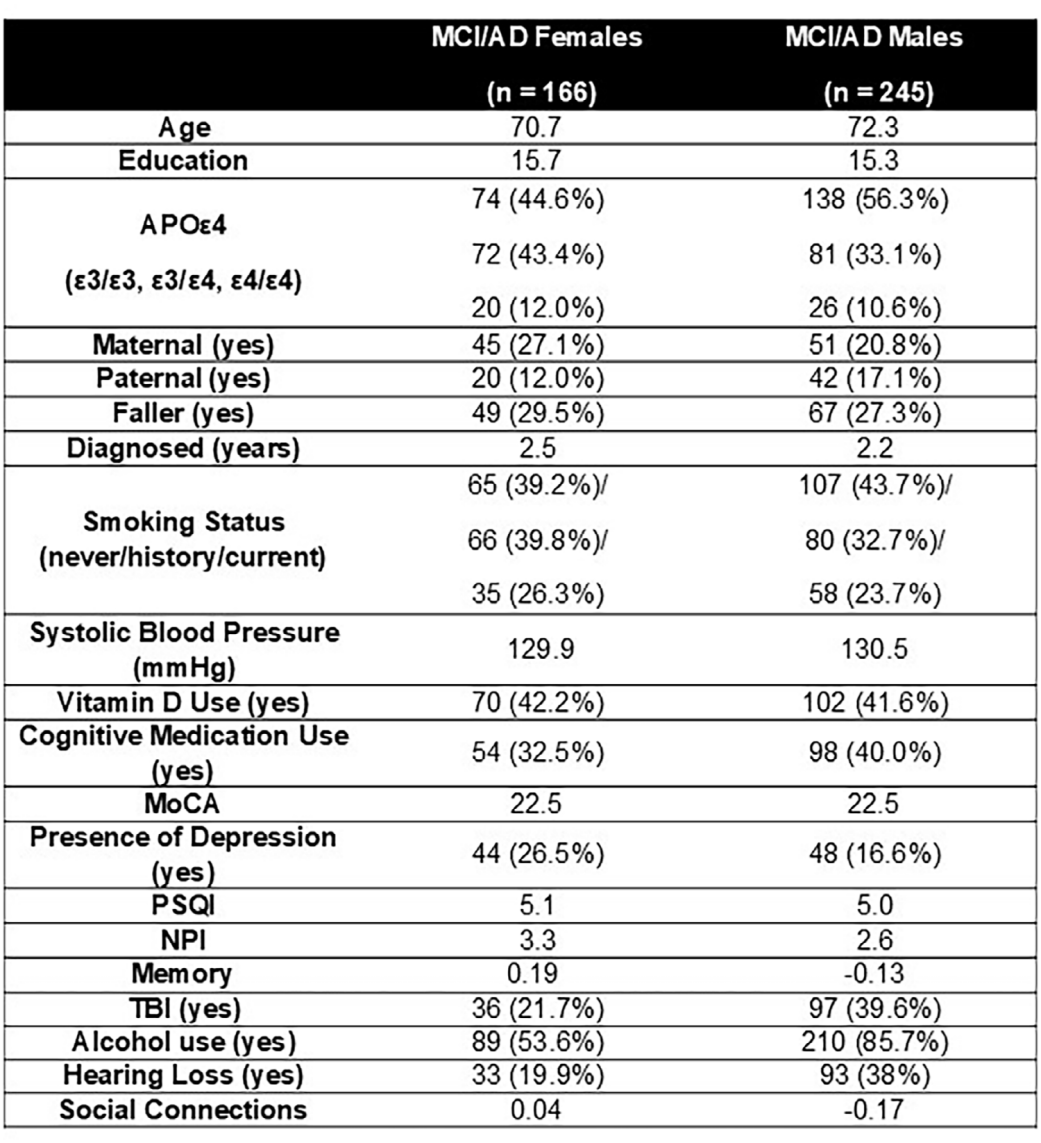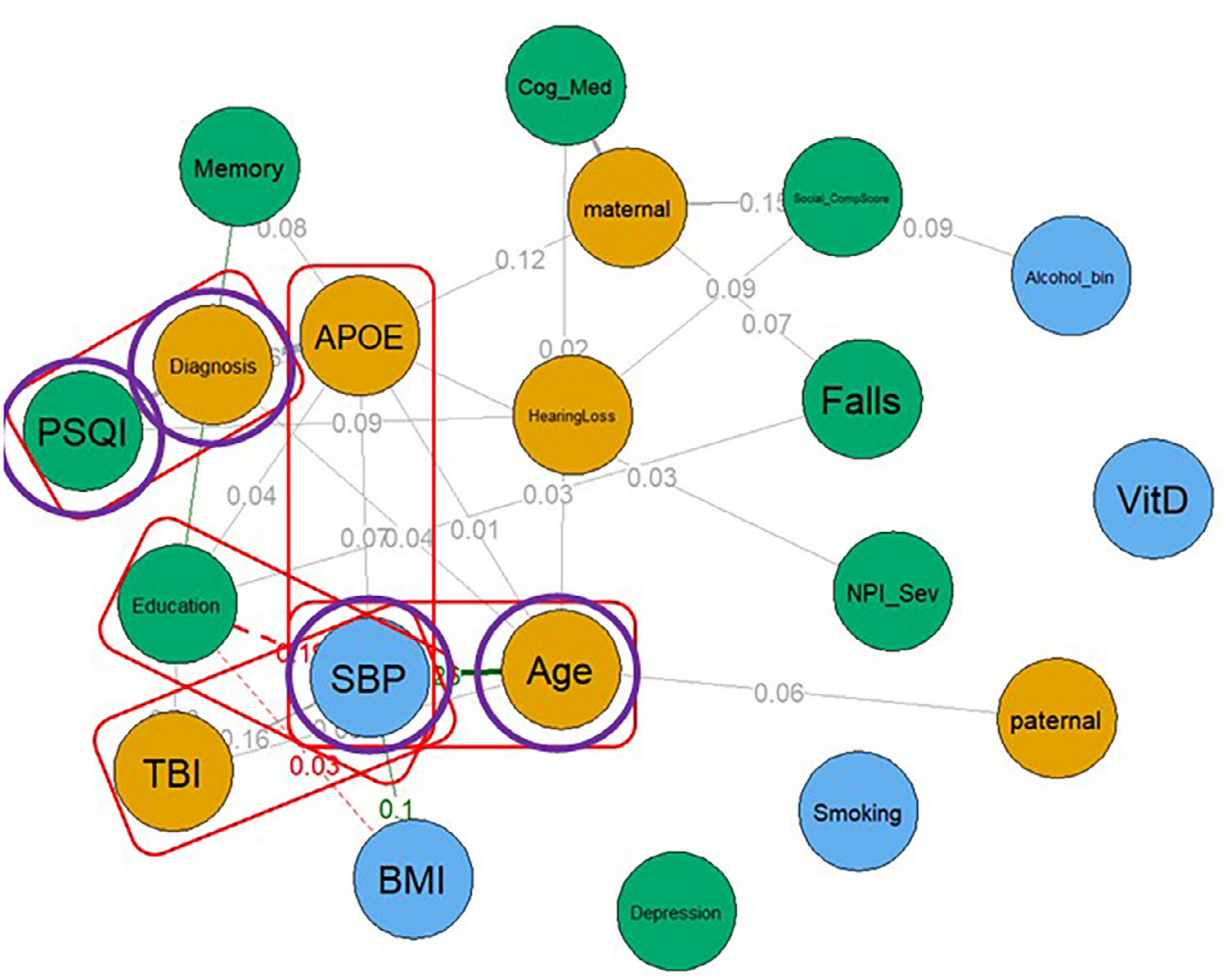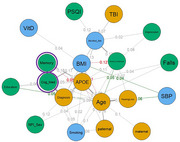# Exploring sex‐specific risk factors in cognitive decline: a network analysis of modifiable and non‐modifiable determinants

**DOI:** 10.1002/alz70860_101074

**Published:** 2025-12-23

**Authors:** Brittany Intzandt, Joel Ramirez, Benjamin Lam, Mario Masellis, Christopher JM Scott, Gillian Einstein, Louis Bherer, Sandra E. Black, Ontario Neurodegenerative Disease Research Initiative Researchers and Canadian Consortium for Neurodegeneration in Aging Researchers

**Affiliations:** ^1^ Sunnybrook Research Institute, Toronto, ON, Canada; ^2^ Dr. Sandra E. Black Centre for Brain Resilience and Recovery, LC Campbell Cognitive Neurology, Hurvitz Brain Sciences Program, Sunnybrook Research Institute, University of Toronto, Toronto, ON, Canada; ^3^ University of Toronto, Toronto, ON, Canada; ^4^ University of Toronto Scarborough, Toronto, ON, Canada; ^5^ Heart and Stroke Foundation Canadian Partnership for Stroke Recovery, Toronto, ON, Canada; ^6^ LC Campbell Cognitive Neurology Research Unit, Sunnybrook Research Institute, Toronto, ON, Canada; ^7^ Sunnybrook Health Sciences Centre, Toronto, ON, Canada; ^8^ Division of Neurology, Department of Medicine, Sunnybrook Health Sciences Centre, Toronto, ON, Canada; ^9^ Cognitive and Movement Disorders Clinic, Sunnybrook Health Sciences Center, Toronto, ON, Canada; ^10^ Hurvitz Brain Sciences Program, Sunnybrook Research Institute, Toronto, ON, Canada; ^11^ Linkoping University, Linkoping, Sweden; ^12^ Rotman Research Institute, Toronto, ON, Canada; ^13^ Université de Montréal, Montréal, QC, Canada; ^14^ Montreal Heart Institute, Montréal, QC, Canada; ^15^ Hurvitz Brain Sciences Program, Toronto, ON, Canada; ^16^ Toronto Dementia Research Alliance, Toronto, ON, Canada

## Abstract

**Background:**

Dementia incidence is projected to nearly triple by 2050 worldwide^1^, posing unique challenges to healthcare systems. Identifying non‐modifiable and modifiable risk factors (RF) is crucial, including sex‐specific factors, given the higher prevalence of dementia among females (60%)^2^. This study employed to network analysis to examine common RF for dementia, as identified in the Lancet Commission^3,4^ in individuals with cognitive decline (CD) comparing sex‐specific networks to identify unique RF and interactions.

**Method:**

411 individuals with CD were included (mild cognitive impairment and Alzheimer's dementia) from the Ontario Neurodegenerative Disease Research Initiative (30% of participants) and Canadian Consortium for Neurodegeneration in Aging (52% female – Table 1). A network analysis^5^ was used to examine non‐modifiable (e.g., age), modifiable (e.g., sleep, alcohol use) and cognitive outcomes. Sex‐specific networks were created and compared, node centrality was investigated to determine the relative importance of each RF.

**Result:**

No statistical difference in network structure between CD males and females was observed (M = 0.221; *p* = 0.256). The male network did have higher global strength than the females’, indicating greater connectivity among all nodes (S = 0.688; *p* = 0.044)[Figure 1&2). In the female network, nodes that were central (greatest impact on network structure) were systolic blood pressure, diagnosis (mild cognitive impairment vs Alzheimer's disease), age and sleep quality, while memory and cognitive medication use were central nodes for males (*p* < 0.05). Specific edges, such as the relationship between systolic blood pressure and other RF (Figure 1 and 2), were significantly different between networks

**Conclusion:**

Our findings reveal unique sex‐specific network patterns of RF for CD, emphasizing the importance of sex‐disaggregated analyses. Although the overall structure of the networks were not statistically different, key differences in patterns for the edges and nodes emerged, with males demonstrating higher connectivity among RF, and thus a more closely related network of RF. These findings point to significant gaps in our understanding of sex‐specific RF for CD and highlight the need for further investigation to disentangle these complex relationships. Future work should integrate biomarkers, such as neuroimaging to further explore how sex‐specific RF influence dementia risk.